# Supplementary material for: Mental health challenges and resilience strategies of Indigenous youth citizen scientists living in rural areas during COVID-19 school closures
Source: PLOS Ment Health. 2025 Jun 4;2(6):e0000256. doi: 10.1371/journal.pmen.0000256 (PMC12798461; doi:10.1371/journal.pmen.0000256)
Supplement: S1 Appendix — (DOCX) [file pmen.0000256.s001.docx]

**S1 APPENDIX**

**FOCUS GROUP TEMPLATE**

| **Beginning of the COVID-19 pandemic**  **Winter 2021** | **During the COVID-19 pandemic**  **Summer 2021** |
| --- | --- |
| **Part I**  The first few questions we will discuss are about the key challenges faced by the students concerning the COVID-19 pandemic in the past seven or eight months.  **Note:** Mid of March 2020 – September 2020-School closure due to COVID-19. | This is a follow-up discussion with youth citizen scientists to understand how they adapt to online learning during the COVID-19 pandemic.  Initially, citizen scientists discuss about COVID-19 pandemic and the implementation of safe school reopening policies.  **Note**: October, 2020 School reopened and adopted online mode of engagement. |
| **School Closure Challenges** | **COVID-19 challenges** |
| 1. How has COVID-19 affected your life in general?  - **Probe**: Think about the changes at school, home, and in your community in general. / How do you feel about the changes that have happened in the past 7 or 8 months? | 1. How do you feel in general these days?  - **Probe**: Have there been any considerable changes that affected your daily life? /Think about the changes at school, home, and in your community in general.  1. How are you managing the new “normal” situation in your school?  - **Probe:** How do you feel about learning in online vs on-school classes? / How do you cope with your online learning? |
| **Health during COVID-19** | **Coping Strategies** |
| 1. How do you think COVID-19 pandemic affected your health?  - **Probe:** Are you doing the same amount of physical activity that you did before COVID- 19? Have you been more or less anxious etc? | 1. How has COVID-19 affected your life in general (studies/mental health/physical activity)? How do you cope with social distancing?  - **Probe**: Do you want to share any events or happenings that affected your life during the COVID-19 pandemic? |
| **Academic Challenges** | **Academic Challenges** |
| 1. Tell us more about the difficulties in doing schoolwork.  - **Probe**: Do you have a computer to work from home? Do you have WIFI at home? If no, how do you access Internet? | 1. In our previous discussion, you pointed out some problems with online learning. Do you think that those problems are now being solved?   **Probe**: Do you have any problems taking online classes? If yes, what are some of these problems?   1. *Communication*: Do you experience any problems in communicating with your educators or school? If yes, what are some of these problems? |
| **Support** |  |
| 1. Whom did you go to get support when you faced difficulties during the past 7-8 months?  - **Probe**: Who helped you out or was easy to reach? |  |
| **Future Concerns** |  |
| 1. What are your future concerns about the COVID-19 pandemic? |  |
| **PART B**  *Next, we will discuss some questions to understand how the culture, active living and mental health help to overcome the COVID-19 pandemic.* |  |
| Culture *Culture means where you are from, teachings from your family and community, and our history.*   1. Do you think your culture has played a role in helping you to cope with the COVID-19 pandemic?  - **Probe**: has your culture helped you during the pandemic? |  |
| **Active Living**   1. What are some ways that people keep active in your community during the COVID-19 pandemic?  - **Probe**: what type of activities do youth in your community engaged in during the school closure? | 1. How is your health? Do you worry about your health in general due to this pandemic?  - **Probe**: Do you engage in any activities to keep yourselves motivated, like physical activity? |
| Mental Health  1. Most of us have probably heard of the term, ‘Mental Health’, what does mental health mean to you?  - **Probe**: What you did to make yourselves feel happy or peaceful in the COVID-19 pandemic? | **Mental Health**   1. Are you aware of the mental health support available through the school? If yes, have you accessed it or intend to access it? If no, what are the reasons for not accessing mental health support? |
| 1. **Final Question -**Is there something the school could help you with to manage your stress or help you to be more active during the pandemic? |  |
