# Supplementary material for: Mental health challenges and resilience strategies of Indigenous youth citizen scientists living in rural areas during COVID-19 school closures
Source: PLOS Ment Health. 2025 Jun 4;2(6):e0000256. doi: 10.1371/journal.pmen.0000256 (PMC12798461; doi:10.1371/journal.pmen.0000256)
Supplement: Attachment — Submitted filename: Response to Reviewers.docx [file pmen.0000256.s003.docx]

**Response to Reviewers**

Karli Montague-Cardoso
Executive Editor
PLOS Mental Health

Journal Requirements:

1. Please include a complete copy of PLOS’ questionnaire on inclusivity in global research in your revised manuscript. Our policy for research in this area aims to improve transparency in the reporting of research performed outside of researchers’ own country or community. The policy applies to researchers who have travelled to a different country to conduct research, research with Indigenous populations or their lands, and research on cultural artefacts. The questionnaire can also be requested at the journal’s discretion for any other submissions, even if these conditions are not met.  Please find more information on the policy and a link to download a blank copy of the questionnaire here: <https://journals.plos.org/plosmentalhealth/s/best-practices-in-research-reporting>. Please upload a completed version of your questionnaire as Supporting Information when you resubmit your manuscript.

**Response:** Thank you. We have now attached the completed ‘Inclusivity in Global Research’ questionnaire to the submission.

2. Please amend your detailed Financial Disclosure statement. This is published with the article. It must therefore be completed in full sentences and contain the exact wording you wish to be published.

The Financial Disclosure statement has been revised as per journal guidelines.

i. Please clarify all sources of funding (financial or material support) for your study. List the grants (with grant number) or organizations (with url) that supported your study, including funding received from your institution.

ii. State the initials, alongside each funding source, of each author to receive each grant.

iii. State what role the funders took in the study. If the funders had no role in your study, please state: “The funders had no role in study design, data collection and analysis, decision to publish, or preparation of the manuscript.”

iv. If any authors received a salary from any of your funders, please state which authors and which funders.

**Response:** This study is funded by the Canadian Institutes of Health Research (CIHR) Project Grant #153226, the Saskatchewan Health Research Foundation (SHRF) Patient Oriented Research Leader Award, and the Canada Research Chair in Digital Health for Equity awarded to TRK. The funders had no role in study design, data collection and analysis, decision to publish, or preparation of the manuscript.

3. Please make sure the funding information on the submission form matches your financial disclosure statement. Please indicate by return the full and correct funding information for your study and confirm the order in which funding contributions should appear. Please be sure to indicate whether the funders played any role in the study design, data collection and analysis, decision to publish, or preparation of the manuscript.

**Response:** Thank you.

4. Please insert an Ethics Statement at the beginning of your Methods section, under a subheading 'Ethics Statement'.

**Response:** An ethics statement has now been added as a new methods subsection (2.1).

5. Please provide separate figure files in .tif or .eps format.

For more information about figure files please see our guidelines:

<https://journals.plos.org/mentalhealth/s/figures>

<https://journals.plos.org/mentalhealth/s/figures#loc-file-requirements>

**Response:** Thank you. Figures have now been uploaded as per formatting guidelines.

6. Tables should not be uploaded as individual files. Please remove these files and include the Tables in your manuscript file as editable, cell-based objects. For more information about how to format tables, see our guidelines:

<https://journals.plos.org/mentalhealth/s/tables>

**Response:** Thank you. Tables have now been uploaded as per the suggested guidelines.

7. In the online submission form, you indicated that “The data supporting the findings of this study are available upon request from the corresponding author.”

All PLOS journals now require all data underlying the findings described in their manuscript to be freely available to other researchers, either

1. In a public repository,

2. Within the manuscript itself, or

3. Uploaded as supplementary information.

This policy applies to all data except where public deposition would breach compliance with the protocol approved by your research ethics board. If your data cannot be made publicly available for ethical or legal reasons (e.g., public availability would compromise patient privacy), please explain your reasons by return email and your exemption request will be escalated to the editor for approval. Your exemption request will be handled independently and will not hold up the peer review process but will need to be resolved should your manuscript be accepted for publication. One of the Editorial team will then be in touch if there are any issues.

**Response:** The study is part of the Smart Platform, a citizen science and mHealth initiative for ethical surveillance, integrated knowledge translation, and policy and real-time interventions. As this study contains sensitive data such as time-stamped location of citizen scientists, data requests should be sent to the University of Regina’s Research Ethics Board at [research.ethics@uregina.ca](mailto:research.ethics@uregina.ca)

8. Some material included in your submission may be copyrighted. According to PLOS’s copyright policy, authors who use figures or other material (e.g., graphics, clipart, maps) from another author or copyright holder must demonstrate or obtain permission to publish this material under the Creative Commons Attribution 4.0 International (CC BY 4.0) License used by PLOS journals. Please closely review the details of PLOS’s copyright requirements here: PLOS Licenses and Copyright. If you need to request permissions from a copyright holder, you may use PLOS's Copyright Content Permission form.

Please respond directly to this email or email the journal office and provide any known details concerning your material's license terms and permissions required for reuse, even if you have not yet obtained copyright permissions or are unsure of your material's copyright compatibility.

**Response:** Thank you for your comments and the opportunity to clarify.

We confirm that **Figure 2**, which was included in our submission, is **adapted from a previously published open-access article** in JMIR mHealth and uHealth (<https://mhealth.jmir.org/2019/8/e14056/>). This source is published under the **Creative Commons Attribution 4.0 International (CC BY 4.0) license**, which is fully compatible with PLOS’s copyright requirements. Accordingly, we have provided full citation details and appropriate license attribution in the figure legend as follows:

Adapted from: Katapally TR. The SMART framework: integration of citizen science, community-based participatory research, and systems science for population health science in the digital age. JMIR mHealth and uHealth. 2019 Aug 30;7(8):e14056. Licensed under CC BY 4.0.

We also confirm that no copyrighted material under incompatible licensing has been included in the manuscript. Figure 2 was created by TK, while Figures 1 and 3–9 were created by PK. All figures originate from the authors or have been developed in accordance with appropriate licensing and copyright requirements.

Potential Copyright Issues:

a. We do not publish any copyright or trademark symbols that usually accompany proprietary names, eg (R), (C), or TM (e.g. next to drug or reagent names). Therefore please remove all instances of trademark/copyright symbols throughout the text, including (“OCAP®”) on page 11.

**Response:** We have **removed all instances of trademark and copyright symbols** (e.g., “OCAP®”) throughout the manuscript text in accordance with PLOS formatting requirements.

Please review your reference list to ensure that it is complete and correct. If you have cited papers that have been retracted, please include the rationale for doing so in the manuscript text, or remove these references and replace them with relevant current references. Any changes to the reference list should be mentioned in the rebuttal letter that accompanies your revised manuscript. If you need to cite a retracted article, indicate the article’s retracted status in the References list and also include a citation and full reference for the retraction notice.

**Response:** We have reviewed the **reference list** and confirm that it is complete and up to date. No retracted articles have been cited. If any article had been retracted, we would have noted the retracted status in both the in-text citation and full reference, including citation of the retraction notice, per PLOS guidelines.

Thank you for the comment. We have carefully reviewed the reference list and confirm that it is complete, correct, and does not include any retracted articles.

Additional Editor Comments (if provided):


[Note: HTML markup is below. Please do not edit.]

Reviewers' comments:

**Reviewer's Responses to Questions**

**Comments to the Author**

1. Does this manuscript meet PLOS Mental Health’s [publication criteria](https://track.editorialmanager.com/CL0/https:%2F%2Fjournals.plos.org%2Fmentalhealth%2Fs%2Fcriteria-for-publication/1/010f0195a7a9b1eb-825cb910-7bcd-4f67-a1d3-e433c8d39d56-000000/UknIjQi8ItQJLfbsxrULR1FvRLeQnbAH54gX5e1vNx4=202)? Is the manuscript technically sound, and do the data support the conclusions? The manuscript must describe methodologically and ethically rigorous research with conclusions that are appropriately drawn based on the data presented.

Reviewer #1: Partly

Reviewer #2: Yes

2. Has the statistical analysis been performed appropriately and rigorously?

Reviewer #1: N/A

Reviewer #2: N/A

3. Have the authors made all data underlying the findings in their manuscript fully available (please refer to the Data Availability Statement at the start of the manuscript PDF file)?

The [PLOS Data policy](https://track.editorialmanager.com/CL0/https:%2F%2Fjournals.plos.org%2Fmentalhealth%2Fs%2Fdata-availability/1/010f0195a7a9b1eb-825cb910-7bcd-4f67-a1d3-e433c8d39d56-000000/DuoKYLe35VJqRROWt7bglQGi4ju3aOyLzH2raLGKCW0=202) requires authors to make all data underlying the findings described in their manuscript fully available without restriction, with rare exception. The data should be provided as part of the manuscript or its supporting information, or deposited to a public repository. For example, in addition to summary statistics, the data points behind means, medians and variance measures should be available. If there are restrictions on publicly sharing data—e.g. participant privacy or use of data from a third party—those must be specified.

Reviewer #1: Yes

Reviewer #2: No

4. Is the manuscript presented in an intelligible fashion and written in standard English?

PLOS Mental Health does not copyedit accepted manuscripts, so the language in submitted articles must be clear, correct, and unambiguous. Any typographical or grammatical errors should be corrected at revision, so please note any specific errors here.

Reviewer #1: Yes

Reviewer #2: Yes

5. Review Comments to the Author

Please use the space provided to explain your answers to the questions above. You may also include additional comments for the author, including concerns about dual publication, research ethics, or publication ethics. (Please upload your review as an attachment if it exceeds 20,000 characters)

Reviewer #1: PNEM-D-24-00569
Mental Health Challenges and Resilience Strategies in Rural Areas during COVID-19 School Closures: Indigenous Youth Citizen Scientists Insights from a Natural Experiment
Overall comment
This is an interesting article. It not only focuses on mental health challenges of COVID 19, but takes it a step further to investigate the resilience of learners through the pandemic. Even though authors did a good job, I suggest a few changes that would strengthen their work. The article would also benefit from a language editor.

Title
The title does reflect information found in the article. However, consider swapping a few words to make it simpler and easier to comprehend. eg Mental health challenges and resilience strategies of Indigenous Youth Science Citizens Scientists living in rural areas during COVID-19 school closures.

**Response**: Thank you for this helpful suggestion. We have revised the title to enhance clarity and readability. Additionally, the manuscript has been reviewed by a language editor, and appropriate edits have been made accordingly.

Abstract
Even though all elements required in an abstract are included, I would suggest that authors trim it down and only leave information that speaks to the following; aim of the article, one or two lines that show the importance of this aim, methods of data collection used (including demographics such as sample size, age range). Findings (themes), then briefly situate findings in literature (one or two lines), why your study is crucial given these findings, who will/can benefit from your research (optional). This would reduce the length and increase its effectiveness.

**Response:** Thank you for this constructive feedback. In response, we have revised the abstract to focus more concisely on the core elements outlined.

Background
I suggest you strengthen how you transition from discussing mental health challenges of COVID 19, to coping mechanisms and how you position your study. For instance, you could follow your discussion of challenges with a discussion of children and youth’s coping mechanisms (in general), then narrow it down to Indigenous youth and children. This clearly defined funnel approach would then position your study better.

**Response:** Thank you for this insightful suggestion. In response, we have revised the background section to adopt a clearer narrative.

Methodology
While authors do make reference to sampling and focus group discussions, there is a need for a more thorough description of their decisions related to sampling, and focus group discussions in 2.6.

**Response:** Thank you for your comment. We have revised Section 2.6 to include a clearer rationale for the sampling strategy and expanded details on how the focus group discussions were designed and conducted, including co-development of questions, participant selection, and facilitation format.

Results
Here are a few copy and paste tweaks that would strengthen your findings.

Theme 1
Consider only using two quotes instead of 3 if they all advance a similar point. Reconsider YCS3‘s quote under ‘females missing social interaction’. It does not support that point. Also provide data that supports this next point or consider deleting it. “However, some of the concerns are similar for both genders”.

Theme 3
Consider placing the first two quotes immediately after “They most frequently leaned on family, friends, cousins, and partners”. Then move your sentence of “However, it is worth noting…” directly above YCS 6’s quote. Consider moving some quotes from theme 7 (coping mechanisms) to this theme because they also refer to support. For instance, playing and taking walks with family and friends was a facilitative to both males and females’ resilience. Then rethink your statement that males did not rely on family support.

Theme 4- consider swapping theme 4 with theme 3 so that you group risk themes together and resilience themes together.

Theme 5
Theme 5 is still a form of support. Rethink your layout. For instance- I would make support the main theme. Then have subcategories: a) family and friends support b) cultural support

Theme 5, Support, comprises two sub-categories: (a) support from family and friends, and (b) cultural support, each described in detail below.

**Follow up FG**
Theme 2- consider starting YCS4 quote from “and my teacher…”. Add a quote or 2 that support this statement…”some YCSs managed to finish their school tasks ahead of schedule”.

**Response:** Thank you for your detailed and constructive feedback on the Results section. We have carefully reviewed and addressed your suggestions. Revisions include refining the number and placement of quotes, ensuring each supports the relevant theme, rewording or removing unsupported statements, and reorganizing the theme sequence to clearly distinguish between risk and resilience themes. We have also restructured Theme 5 to present “Support” as a main theme with appropriate sub-categories and adjusted follow-up focus group quotes for clarity and alignment with the supporting statements.

**Discussion**
Consider removing the first paragraph and start the section strong with paragraph 2.
Even though discussing policy implications is important, consider compressing this information into one or two paragraphs. Then write a separate article that delves deeper into policy implications.

**Response:** Thank you for your thoughtful recommendation. We have removed the initial paragraph to strengthen the opening of the section and revised the structure for improved focus and flow. The policy implications have been condensed into two concise paragraphs, as suggested, to better align with the scope of the current article. We appreciate your suggestion to explore policy implications further in a separate publication and will consider this for future work.

Reviewer #2: It was a pleasure reviewing your work entitled: “Mental Health Challenges and Resilience Strategies in Rural Areas during CO School Closures: Indigenous Youth Citizen Scientists Insights from a Natural Experiment,” a study that explores the perspective of Indigenous youths’ mental health impact from the COVID-shut down. The study has merit for change and insights that centers Indigenous youth voices but my main concern is in your methods, its delivery, language and coherency with the claims of citizen science.

Firstly, don’t you think the youth involved as co-researchers in your work should be cited as co-authors also? In the spirit of citizen science?

**Response**: Thank you for this thoughtful question. We fully acknowledge the essential contributions of the youth citizen scientists who participated in this study. Their perspectives informed every stage of the research from conceptualization and identifying community health priorities to shaping study materials and participating in focus group discussions. The Citizen Scientist Advisory Council served as a vital bridge between the research team and the community, ensuring the study remained grounded in local context and culturally relevant values.

However, in accordance with ethical research practices and in consultation with the participating communities, this study was conducted with a commitment to preserving participant anonymity. The decision not to include youth Citizen Scientists as named co-authors was made in alignment with the community’s expressed preference to maintain confidentiality, especially given the sensitive nature of the study and the small population size involved. Rather than individual recognition, the community prioritized ensuring that the findings be used to inform school policy and improve mental health supports for Indigenous youth.

Although not listed as co-authors, the youth Citizen Scientists and community members are formally acknowledged in the manuscript. Additionally, the research team has engaged in ongoing knowledge translation by sharing written updates and findings with participants throughout the study, demonstrating our continued commitment to reciprocal and respectful collaboration.

Abstract
Apart from the tautology in the description of your study design “qualitative, pre-post, quasi-experimental citizen science study involved eight Indigenous youth citizen scientists (YCS),” you conclusion is lacking in summarizing the essence of the study. Your conclusion should summarize key findings related to your study objective, provide practical implications and suggest future directions(if any). Rather you focus on something how the methodology demonstrates something unrelated to your primary study objective, which is not an answer to the original question set out to explore. Or is this a methods paper? Except you wanted to show how using an hybridized study design as you did, I suggest to stick to the original objective and other sections should logically flow out of that.

**Response:** Thank you for your insightful feedback. We have revised the abstract to address the tautology in the study design by removing redundant phrasing. The conclusion has been rewritten to align with the study’s primary objective. It now summarizes key findings, outlines practical implications, and suggests future directions. These changes ensure the abstract flows logically from the study objective and clarifies that the focus is on the findings rather than the methodology.

**Introduction/methods**

Well articulated and executed introduction starting the problem theme from a global stage narrowing to children and youth. But nothing said (with regards to statistical figures and prevalences)about Indigenous youth and how it impacted them relative to non-Indigenous youth (given that your study wants to focus on Indigenous).

The abrupt jump from coping strategies to specialized mental health services makes readability difficult. If your aim is to emphasize the value of community-engaged research that was not a good work through to that point.

I also have some misgivings around the use of “Indigenous” as a broad-based terminology. Also, the claim they were citizen scientist (a term used by public health researchers to describe partners in a participatory research setting). Whie that is not a hill to die on, consider defining your use of the terminology.

Also per TCPS chapter 9 rules (which you cited), and the Truth and Reconciliation Commission calls to action, research involving Indigenous people in Canada must reflect their culture and values and co-created/driven by them in the spirit of self-determination and self-governance respecting their rights to data sovereignty. I fear I don’t see enough of these reflected in your work, how the relationships were built or how cultural safety was honored in including Elders and traditional knowledge holders in the processes of your community-engaged work. Granted your section overviewing the SIY initiative touched on them but you spend a huge portion is dedicated to describing/explaining the methodology (CS and TES) from a top-down rather than a bottom-up culturally grounded approach, makes it unrepresentative of true engagement or cultural humility/sensitivity. A section of your work should focus on the process of engagement rather than lumping them in between sections or using general statements like: “The team has built strong partnerships with participating Indigenous communities and adopted the Smart Framework…”

If community was engagement the language should reflect that is all I am saying. Who decided on the choice of study design, design of questions, carried out analysis, and interpretation, etc. state these for readers to appreciate the rigor of your relationship building process. The smart framework in my opinion read top down (designed by the research team) and forced on the youth participants rather than co-designed by and with them as it should in a true CS methodology.

For the sake of readers misconstruing your intent, it would be important to state how the youth were engaged from the beginning of this work. Often times we cite work involving Indigenous youth as youth-led or youth-engaged and the level of engagement is nothing short of extractive or helicopter research. If youth were truly engaged provide justification for why the team chose to carry interviews virtually (that’s not signifying true engagement, even though I understand it was around the time of COVID). I want more depth in explaining or justifying your claims is all I mean. I see the intent but I don’t know that others might the way you word some of the sections outlining engagement science.

**Response:** Thank you for your thoughtful and detailed feedback. We agree that incorporating these details strengthens the study narrative and more clearly articulates the collaborative research conducted with the community. To address your comments, we have added statistical context on Indigenous youth to better frame the study’s relevance, clarified our use of the term “citizen scientist,” and improved transitions for better flow in paragraph 3 and the final paragraph of the Introduction.

We revised the methods and background sections to explicitly describe how Elders, youth, and educators co-developed the study design, questions, and analysis (see methods 2.2 section 3 paragraph). We also added a dedicated section on the engagement process to reflect bottom-up, culturally grounded participation and to align with TCPS2 and the TRC Calls to Action as a new Methods subsection 2.1. Moreover, the rationale for virtual engagement was expanded to reflect community-informed decisions during COVID-19 in section 2.3.

6. PLOS authors have the option to publish the peer review history of their article ([what does this mean?](https://track.editorialmanager.com/CL0/https:%2F%2Fjournals.plos.org%2Fmentalthealth%2Fs%2Feditorial-and-peer-review-process%23loc-peer-review-history/1/010f0195a7a9b1eb-825cb910-7bcd-4f67-a1d3-e433c8d39d56-000000/5mCa3t7dN3s1EXe1or2ZAfF7kCyT1igl168Aw2kEgO0=202)). If published, this will include your full peer review and any attached files.


**Do you want your identity to be public for this peer review?** If you choose “no”, your identity will remain anonymous but your review may still be made public.


For information about this choice, including consent withdrawal, please see our [Privacy Policy](https://track.editorialmanager.com/CL0/https:%2F%2Fwww.plos.org%2Fprivacy-policy/1/010f0195a7a9b1eb-825cb910-7bcd-4f67-a1d3-e433c8d39d56-000000/OwP35qMRWGUFr9RIjFaMbKi_DAMHeBWOJ30MDyuBJBw=202).

Reviewer #1: **Yes:** Dr Nombuso Gama

Reviewer #2: **Yes:** Udoka Okpalauwaekwe

[NOTE: If reviewer comments were submitted as an attachment file, they will be attached to this email and accessible via the submission site. Please log into your account, locate the manuscript record, and check for the action link "View Attachments". If this link does not appear, there are no attachment files.]

While revising your submission, please upload your figure files to the Preflight Analysis and Conversion Engine (PACE) digital diagnostic tool, <https://pacev2.apexcovantage.com/>. PACE helps ensure that figures meet PLOS requirements. To use PACE, you must first register as a user. Registration is free. Then, login and navigate to the UPLOAD tab, where you will find detailed instructions on how to use the tool. If you encounter any issues or have any questions when using PACE, please email PLOS at [figures@plos.org](mailto:figures@plos.org). Please note that Supporting Information files do not need this step.
